# Longitudinal optical coherence tomography indices in idiopathic intracranial hypertension

**DOI:** 10.1038/s41598-024-58865-3

**Published:** 2024-04-14

**Authors:** Rachel Shemesh, Omry Frige, Sharon Garmider, Ruth Huna-Baron

**Affiliations:** 1 Neuro-Ophthalmology Unit, The Goldschleger Eye Institute, Sheba Medical Center, Tel Hashomer, Israel; 2 Faculty of Medicine, Tel Aviv University, Tel Aviv, Israel; 3 Arrow Project, Sheba Medical Center, Tel Hashomer, Israel

**Keywords:** Idiopathic intracranial hypertension, Optical coherence tomography, Nadir retinal nerve fiber layer thickness values, Nadir ganglion cell complex values, Neurology, Diseases, Eye diseases, Optic nerve diseases

## Abstract

Idiopathic intracranial hypertension (IIH) may result in optic nerve fiber loss and even atrophy. The timing of the optical coherence tomography (OCT) indices reaching the lowest point (nadir) and the factors that predict the patient's anatomical outcome are not known. We aimed to determine the timing and the factors that affect nadir retinal nerve fiber layer (RNFL) thickness. The medical records of 99 IIH patients who were treated from December 2009 to January 2020 were retrospectively reviewed. The mean RNFL thickness at presentation was 263.5 ± 106.4 µm. The mean time to nadir was 7.9 ± 6.3 months. The average RNFL and ganglion cell complex (GCC) thickness at the nadir were 92.6 ± 14.5 µm (47% showed thinning) and 77.9 ± 27.8 µm (70% showed thinning), respectively. The Frisén disc edema stage and average RNFL thickness at baseline correlated with a longer time to nadir, (r = 0.28 *P* = 0.003 and r = 0.24, *P* = 0.012, respectively). The nadir average RNFL thickness and the nadir average GCC thickness (r = 0.32, *P* = 0.001, r = 0.29, *P* = 0.002, respectively) correlated with the baseline visual field mean deviation. The final anatomical outcome of IIH episodes in this study resulted in RNFL and GCC thinning. The time to RNFL nadir and its values correlated with IIH severity at presentation.

## Introduction

Idiopathic intracranial hypertension (IIH) is a condition of increased intracranial pressure of unknown cause^1^. The keystone of treatment is weight reduction accompanied by pharmacological or surgical therapies^2^. Untreated or sub optimally treated IIH may result in optic nerve atrophy, which may cause permanent loss of vision^3^. There are only few reports of optical coherence tomography (OCT) indices in IIH patients. Farahat et al. measured retinal nerve fiber layer (RNFL) thickness by OCT in IIH patients and found that RNFL thickness can provide important information on retinal axonal loss^4^. Other studies have found that eyes with resolved chronic papilledema show a significant reduction in macular thickness^5^, and macular thickness measurements can be used to monitor the amount of ganglion cell complex loss (GCC)^6^. However, the precise timing of the appearance of the lowest point (nadir) OCT indices is not known.

To the best of our knowledge, the factors that predict the outcome of IIH patients as reflected in nadir OCT measurement values have not been previously studied. The aim of this study was to determine the timing of nadir OCT results and the factors that affect the nadir RNFL and GCC thickness values.

## Methods

### Participants

**Figure 1:**
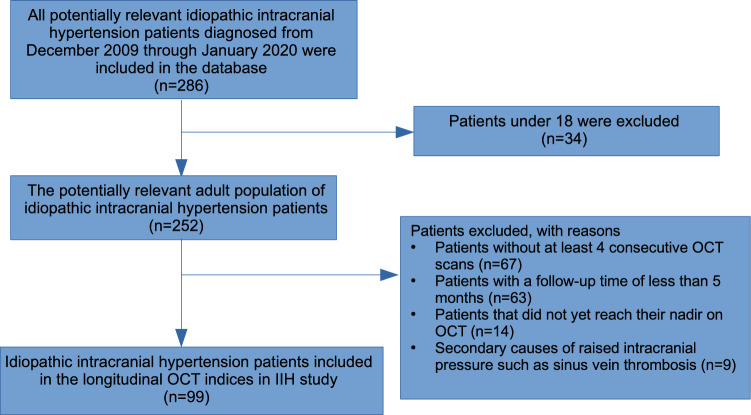
Summary of all potentially suitable idiopathic intracranial hypertension patients retrieved from the database.

The inclusion and exclusion criteria adopted were the same as for most IIH studies^2,7–9^. Eligible participants were 18 years of age or older who had been diagnosed with IIH according to the modified Dandy criteria^10^ and were treated in our institution from December 2009 to January 2020 with at least 5 months of follow-up and at least 4 consecutive OCT scans. The first OCT was performed within 10 days of presentation and within one week of the lumbar puncture. Exclusion criteria were applied to help refine the data and ensure against miscoding secondary causes of raised intracranial pressure, such as brain tumors and sinus vein thrombosis. In addition, patients with optic disc drusen or refractive error of myopia > − 5.00 or hyperopia >  + 5.00 were also excluded. The medical records of all consecutive IIH patients were retrospectively reviewed. Recurrence was defined as disc edema stage 1 or higher after documentation of edema resolution. A total of 286 patients were identified, of whom 187 were excluded (Fig. 1).

### Data collection

Data on demographics, presenting symptoms, past ocular history, past medical history, and medication use were collected. The following data were also collected at presentation and at the appearance of nadir OCT measurements: body mass index (BMI), visual acuity (VA), color vision (CV), visual field (VF) mean deviation (MD), OCT RNFL, and GCC values. Best corrected VA was determined by the Snellen chart, and Snellen VA was converted to a logMAR value. CV was tested with the Hardy Rand Rittlers (HRR) pseudochromatic plates and documented as number of plates identified out of 6 control plates (plate #5 to plate #10). VFs were examined by the Humphrey automated static perimetry (HVF) 24-2, and the MD was recorded. The average RNFL thickness of the optic disc and GCC thickness values were measured with the Cirrus HD-OCT device (Zeiss Meditec, Dublin, CA USA. The findings of neuroimaging studies, including computed tomography (CT), and CT venography (CTV) and magnetic resonance imaging (MRI) and MR Venography (MRV), lumbar puncture (LP) opening pressure (OP) values, and management were documented as well.

The appearance of a nadir value of the average RNFL and GCC values was determined as the first OCT scan that demonstrated the lowest average RNFL and GCC thickness values that were maintained for at least 3 consecutive OCT scans using the RNFL and GCC protocols. The device compares the measurements to the OCT values of a healthy population matched by age and gender. Only good quality scans, defined as scans with signal strength ≥ 6, were used for analysis. The RNFL and GCC thickness values were considered as having become thicker or thinner using the Cirrus HD-OCT normative database. The eye with the worst baseline MD was defined as the study eye for each patient. The worse eye was determined by the higher average RNFL thickness value at presentation in cases where both eyes had similar MD values (i.e., a difference of less than 0.75 dB).

The study followed the Helsinki ethical principles and was approved by the institutional review board of the Sheba Medical Center (SMC 7770-20). All methods were carried out in accordance with relevant guidelines and regulations. The Sheba Medical Center's ethics committee waived informed consent from participants because data were collected retrospectively and held anonymously.

### Statistical analysis

The Kruskal–Wallis test and the Mann–Whitney test were used to compare continuous variables, while Spearman's rank correlation coefficient was used to study the association between continuous variables. The cumulative incidence of disease recurrence was described by 1 minus Kaplan–Meier curves. The length of follow-up was estimated with the reverse sundering method. SPSS software was used (IBM SPSS Statistics, version 25, Armnok, New York, USA 2017).

## Results

### Demographics

Most (89.9%) of the 99 included patients were women, and the cohort's average age at presentation was 29.3 ± 9.2 years (range 18–58 years). A medical history relevant to IIH was noted for 30 patients (30.3%) (Table 1). Five patients (5.1%) have been taking drugs related to secondary IIH (Table 1).

**Table 1:** Baseline characteristics of all potentially suitable patients.

Female sex n (%)	89 (89.9)
Age (y)	
Mean ± SD	29.32 ± 9.2
Range [Min, Max]	[18, 58]
BMI	
Mean ± SD	32.86 ± 5.49
Range [Min, Max]	[22.59, 47.0]
PMH, n, (%)	30 (30)
Migraine	8 (8.1)
Hypertension	4 (4.0)
Psoriasis	4 (4.0)
Diabetes	3 (3.0)
Polycystic ovary syndrome	3 (3.0)
Asthma	3 (3.0)
Celiac disease	3 (3.0)
Depression	3 (3.0)
Fibromyalgia	3 (3.0)
Ocular history, n (%)	31 (37.4)
Mild myopia (up to − 3.00 diopters)	25 (25.3)
History of refractive surgery	4 (4.0)
Mild astigmatism	3 (3.0)
Drugs related to secondary IIH, n (%)	5 (5.1)

*BMI* body mass index, *IIH* idiopathic intracranial hypertension, *SD* standard deviation, *min* minimum, *max* maximum, *PMH* previous medical history.

### Presentation

#### Presenting signs and symptoms

The duration of symptoms before diagnosis was 8.6 ± 7.9 weeks. The symptoms at presentation were headache in 91 patients (91.9%), visual obscurations in 47 (47.5%), tinnitus in 39 (39.4%), and diplopia in 16 (16.2%). The Frisén Scale examination revealed that 11 patients (11.1%) had stage 1disc edema, 39 (39.4%) stage 2, 40 (40.4%) stage 3, and 19 (19.4%) stage 4. The mean VA at presentation was − 0.01 ± 0.09 in logMAR. The mean HRR color test was 5.2 ± 1.3 plates. The MD at presentation was − 7.8 ± 5.3 dB, and the mean LP OP value was 387.6 ± 82.5 mm water. The cohort's mean BMI was 32.9 ± 5.5 kg/m^2^.

### Imaging of patients at presentation

All patients underwent imaging by means of one or more of the imaging modalities, including CT, (77 patients, 77.8%), CTV (70 patients, 70.7%), MRI (55 patients, 55.6%), and MRV (49 patients, 49.5%). All patients had a CTV or MRI with contrast and MRV in addition to CT (77 had CT, among them 70 had also CTV, 33 had an MRI with contrast, and 22 had only MRI and MRV). The following imaging findings were documented in decreasing order: enlarged optic nerve sheath complex (71 patients, 71.7%), enlarged sella turcica (51 patients, 51.5%), and transverse sinus stenosis (45 patients, 45.5%).

### Management of patients

All patients were recommended to follow a weight loss and activity program at presentation. All patients were treated with acetazolamide (Diamox), and the initial recommended dose at presentation was between 500 and 1000 mg daily in 91 (91.9%) cases; the rest received a higher dosage of up to 2 g daily. In addition, 9 patients (9.1%) were treated with furosemide (Fusid) between 20 to 40 mg, and 4 patients (4.0%) were treated with topiramate (Topamax) between 25 and 100 mg. Six patients (6.1%) required optic nerve fenestration in the worse eye as determined by the worst baseline MD or the higher average RNFL thickness value at presentation in cases where both eyes had similar MD values. Four patients (4.0%) required a ventriculoperitoneal shunt, 7 patients (7.1%) underwent diagnostic angiography, and 6 patients (6.1%) underwent venous stenting.

### OCT

The average RNFL and GCC thickness at presentation were 263.5 ± 106.4 µm (range 121–582), and 80.1 ± 11.9 µm (range 50–110), respectively.

### Nadir

The symptoms accompanying nadir OCT findings were as follows: headache (36 patients, 36.4%), tinnitus (27 patients, 27.3%), visual obscurations (7 patients, 7.1%), and diplopia (3 patients, 3.0%). The mean VA at the time of a nadir finding was − 0.009 ± 0.06 in logMAR, and 90 (91%) patients had a VA of 20/20 or better. The mean HRR color test was 5.5 ± 0.8 plates. There was a significant improvement in the CV (p = 0.005). There was an increase in the percentage of patients with VA of 20/20 or better (from 86 to 91%). There was a significant improvement in the MD (from − 7.8 ± 5.3 to − 3.4 ± 4.0 dB, (p < 0.001). The mean BMI was 31 ± 5.6, and it had decreased from baseline in 58% of the patients.

### OCT

The mean gap between the baseline OCT and the nadir OCT finding was 7.9 ± 6.3 months. The mean time between the three consecutive OCT scans was 7.4 ± 2.2 months. The average RNFL and GCC thickness at the time of the nadir result were 92.6 ± 14.5 µm (range 57–129) and 77.1 ± 9.7 µm (range 37–93), respectively. At the time of nadir documentation, 46 (46.5%) of the patients had a thinner average RNFL thickness compared to the Cirrus HD-OCT normative database. Additionally, 69 (69.7%) patients had a thinner average GCC than the normative database. The average ratio of RNFL at nadir detection and the RNFL at presentation (RNFL ratio) was 0.42 ± 0.18.

### Correlation between Nadir OCT outcomes, presenting parameters and disease course

The time to nadir correlated with the Frisén disc edema stage at presentation, r = 0.29 (p = 0.003) and with the baseline RNFL thickness, r = 0.62 (p < 0.001). In addition, a higher baseline LP OP correlated with the average baseline RNFL thickness (r = 0.22, p < 0.023) and with a lower nadir RNFL ratio (r = − 0.28, p = 0.004), (Table 2).

**Table 2:** Spearman rank correlation coefficients for presentation parameters.

Baseline	Time to Nadir R (p value)	Nadir average RNFL thickness R (p value)	Nadir average GCC thickness R (p value)	Ratio of Nadir RNFL average thickness: baseline RNFL average thickness R (p value)
Age	0.08 (0.4)	− 0.09 (0.362)	− 0.02 (0.833)	0.20 (0.12)
BMI	− 0.01 (0.959)	0.01 (0.929)	0.05 (0.591)	− 0.01 (0.887)
Time of symptoms before presentation	− 0.01 (0.900)	− 0.02 (0.805)	− 0.06 (0.544)	0.14 (0.162)
LP OP	− 0.03 (0.737)	− 0.16 (0.111)	− 0.13 (0.202)	− 0.28** (0.004)
Edema stage	0.29**(0.003)	− 0.12 (0.218)	− 0.10 (0.309)	− 0.67** (0.000)

*Time to nadir* time between the baseline OCT and the nadir OCT, *RNFL* retinal nerve fiber layer, *GCC* ganglion cell complex, *Sig.* 2-tailed significance, *BMI* body mass index, *LP OP* lumbar puncture opening pressure.

*Correlation is significant at the 0.05 level (2-tailed). **Correlation is significant at the 0.01 level (2-tailed).

Bold indicates significant values.

### Correlation between OCT Nadir parameters and visual functions

The average nadir RNFL thickness highly correlated with the baseline MD (r = 0.32, p = 0.001). The RNFL ratio also correlated with the baseline VA (r = − 0.19, p = 0.047) and with the baseline MD (r = 0.37, p < 0.001). Additionally, the average nadir GCC thickness correlated with the baseline VA, CV, and VF MD (r = − 0.26, p = 0.006, r = 0.19, p = 0.045, and r = 0.29, p = 0.002, respectively), (Table 3). The nadir OCT RNFL thickness measurements that were within the normal range correlated with significantly higher HRR scores (p = 0.043), and the nadir OCT RNFL values that were within the normal range correlated with higher baseline MD values (p < 0.05). Furthermore, the nadir OCT RNFL thickness values that were thickest as well as those that were within the normal range correlated with higher average nadir GCC thickness values (p = 0.003 and p = 0.041, respectively).

**Table 3:** Spearman rank correlation coefficients of functional parameters.

	Time to Nadir R (p value)	Nadir average RNFL thickness R (p value)	Nadir average GCC thickness R (p value)	Ratio of the Nadir RNFL average thickness:baseline RNFL average thickness R (p value)
Baseline visual acuity	0.03 (0.763)	− 0.08 (0.398)	− 0.26** (0.006)	− 0.19* (0.047)
Baseline HRR	− 0.04 (0.654)	0.15 (0.111)	0.19* (0.045)	0.17 (0.084)
Baseline MD	0.03 (0.767)	0.32** (0.001)	0.29** (0.002)	0.37** (0.000)
Visual acuity at nadir OCT finding	− 0.04 (0.7)	− 0.08 (0.383)	− 0.21* (0.026)	− 0.20 (0.096)
HRR at nadir OCT finding	0.1 (0.326)	0.26** (0.006)	0.18 (0.069)	0.03 (0.767)
MD at nadir OCT finding	0.17 (0.076)	0.24* (0.013)	0.30** (0.002)	0.25** (0.009)
Initial acetazolamide dose	− 0.04 (0.715)	− 0.13 (0.192)	− 0.28** (0.004)	− 0.26** (0.007)
Total acetazolamide dosage	0.37** (0.000)	0.02 (0.85)	0.04 (0.702)	− 0.26** (0.007)

*Time to nadir* time between the baseline OCT and the nadir OCT, *RNFL* retinal nerve fiber layer, *GCC* ganglion cell complex, *Sig.* 2-tailed significance, *HRR* Hardy Rand Rittlers, *MD* mean deviation.

*Correlation is significant at the 0.05 level (2-tailed). **Correlation is significant at the 0.01 level (2-tailed).

Bold indicates significant values.

There was a significant positive correlation between the nadir average GCC thickness and the baseline average GCC thickness (r = 0.47, p < 0.0001). The RNFL ratio and the nadir average GCC thickness correlated highly with the initial dose of acetazolamide treatment (r = − 0.26, p = 0.007 and r = − 0.28, p = 0.004, respectively).

### Follow-up and recurrence episodes of IIH

**Figure 2:**
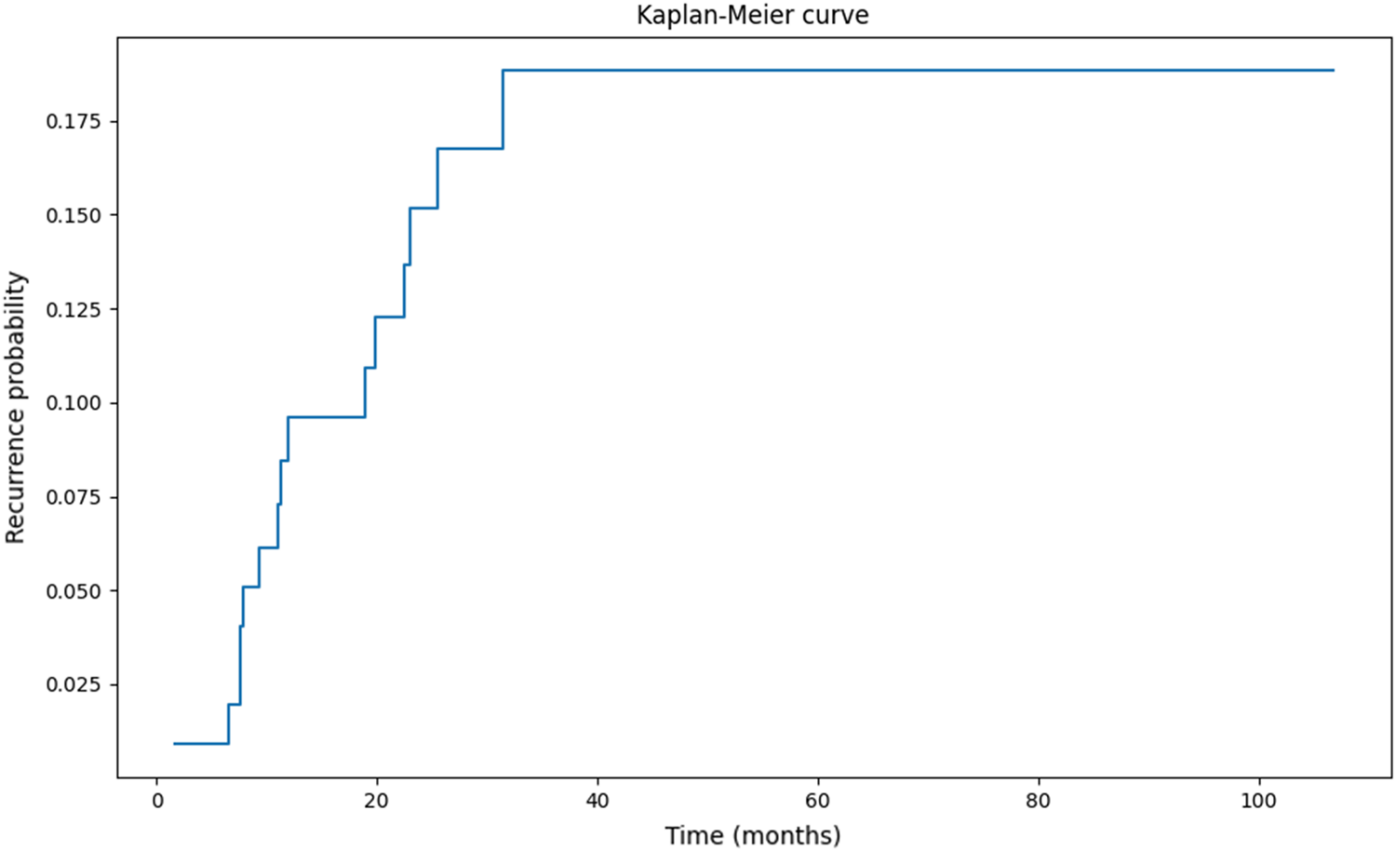
Probability of the recurrence of edematous findings on ocular computerized tomography in idiopathic intracranial hypertension patients.

The median follow-up duration of IIH patients was 40.4 ± 2.4 months, with an interquartile range of 20.7–65 months. Thirteen patients (13.1%) sustained a recurrent episode during this period (Fig. 2).

## Discussion

IIH is considered a benign condition with a favorable outcome^11^. It may, however, be complicated by optic nerve fiber loss and even optic nerve atrophy and may run a chronic and recurrent course of disease in 10–30% of cases^11,12^. The median follow-up duration in our study was 40.4 ± 2.4 months since IIH diagnosis. The relapse rate of our study patients was 13.8%. We demonstrated that a significant number of IIH patients sustained RNFL (47%) and GCC (70%) thinning after a mean of 8 ± 6 months. The time to RNFL nadir as well as the nadir OCT values correlated with IIH severity at presentation.

The percentage of females in our study (89.9%) is similar to the findings in the literature^2,13^. The average age of our patients at presentation was relatively young (29.3 ± 9.2 years), similar to previous studies^2,12,13^. Their high mean BMI at presentation (32.9 ± 5.5 kg/m^2^) was also consistent with earlier reports^2,13^. Furthermore, our patients shared the same common IIH symptoms as those observed by others^3,14^.

Our patients' mean baseline VA was − 0.01 ± 0.09 in logMAR (20/20 on the Snellen chart) and their mean HRR color test result was 5.2 ± 1.3 plates, even in the presence of disc edema. Other studies have similarly noted an intact VA in IIH^15^ as well as good CV^11^. The final anatomical outcome of the IIH episode as defined by OCT resulted in RNFL and GCC thinning, with average RNFL and GCC thickness of 92.6 ± 14.5 µm and 77.9 ± 27.8 µm, respectively. A significant number of our patients (47%) showed RNFL thinning, and 70% showed GCC thinning. This important finding indicates that axonal loss might be more widespread than previously suspected (i.e., reportedly around 33%)^16^, and that it might occur even in apparently effectively treated IIH. The point at which RNFL loss appears on OCT is therefore difficult to define and highly individualized and it was reached after a mean of 8 months since baseline.

The percentage of patients presenting with symptoms was much lower at the appearance of a nadir OCT finding compared to baseline, and the BMI values decreased in 58% of our patients during that time period. There was a concurrent significant improvement in the CV and MD as well as an increase in the percentage of patients with VA of 20/20 or better (91%). The resolution of signs and symptoms correlated with the reduction of disc edema during the follow-up of patients in the IIHTT and the Iowa experience study^2,17^.

The time to RNFL nadir and its values were in correlation with the severity of the condition at presentation in our study. Higher LP OP at baseline was associated with a significantly lower ratio between the nadir RNFL average thickness and the baseline average RNFL thickness. Monteiro et al.^18^ demonstrated that high LP OP values resulted in axonal loss and atrophy in chronic IIH patients. To the best of our knowledge, ours is the first study that demonstrates the quantitative relationship between the two. The results of the IIHTT showed a significant correlation between the average RNFL thickness at baseline and the Frisén grade as well as with the LP OP^19^. In addition, the results of that study found no correlation between the baseline VA or MD and the GCL + IPL thickness^19^. Our study results for the Frisén disc edema stage also correlated with a longer time to a nadir OCT finding.

The average GCC thickness at the time of a nadir OCT result and the RNFL ratio were found to be positively correlated with the VA at presentation. The average nadir GCC thickness was also positively correlated with the CV (HRR score) at presentation, and the nadir average RNFL thickness, the nadir average GCC thickness, and the RNFL ratio were positively correlated with the MD at presentation. In addition, thicker nadir OCT RNFL thickness values in comparison to the Cirrus HD-OCT normative database tended to correlate with higher baseline MD values. Furthermore, normal nadir OCT RNFL thicknesses were correlated with higher HRR scores. These results show that the final anatomical outcome of an IIH episode that results in RNFL and GCC thinning is correlated with the severity of the presentation as well as with the VA, CV, and VF outcomes.

We demonstrated a significant positive correlation between the average baseline GCC thickness and the average nadir GCC thickness. This is in contrast with Huang-Link et al.'s^20^ study which employed the spectral domain OCT and found no change in the GCL–IPL layers irrespective of ICP during a 12-month follow-up period. This difference could be attributed to their small sample size (20 eyes). In our study a significant number of patients showed GCC thinning (69.7%) which correlated with the MD. This finding may suggest irreversible GCC loss from an IIH episode.

In contrast to previous studies emphasizing the importance of aggressive treatment of IIH^13^, in our study, the higher the initial dose of acetazolamide treatment, the lower was the average nadir GCC thickness. This finding could be explained by the greater disease severity in the IIH patients who required a higher initial dose of acetazolamide, as well as by their lower nadir values.

The relapse rate in our long-term study of 99 IIH patients was 13.1%. The relapse rate in our study was lower than the rate of 28% demonstrated by Yri et al.^10^ and 26% by Tovia et al.^11^ who defined relapse as the recurrence of either papilledema or symptoms. The mean observation period in their studies, however, was 74 months, compared to 40.4 months in our study. The relapse rate in our study was similar to that cited in the 10-year observational study by Shah et al.^21^ (15%) in which recurrence was defined as the return of symptoms and the return of previously resolved optic disc edema on clinical examination. We defined relapse as being shown not only by a clinical examination but also by OCT, which is a stricter criterion that increases the validity of establishing recurrence. Taken together, these findings serve to indicate that IIH patients warrant long-term follow-up in order to monitor the occurrence of IIH relapse.

The main limitation of the present study is its retrospective design. Additionally, the baseline GCC measurements were not accurate because of inaccurate segmentation in the edematous state. Moreover, in our study, 71 patients had enlarged optic nerve sheath complexes. All patients in our study had results from at least one brain imaging modality, yet not all patients have completed an MRI. Since CT cannot discriminate the optic nerve from the optic nerve sheaths (as MRI can), theoretically other causes of thick optic nerve sheet complexes cannot be ruled out, such as optic glioma, optic neuritis, or perineuritis. Yet, expansion of the optic complex and optic nerves without enhancement in the presence of an appropriate clinical presentation and normal CSF content rules out inflammatory or tumor involvement in typical cases.

The strength of this study lies in the strict inclusion criteria, the comparatively large sample size, the detailed reports of symptoms, signs, imaging findings, treatment, and the longitudinal follow-up.

In conclusion, this average 40-month follow-up study of 99 IIH patients showed a high rate of RNFL and GCC thinning in IIH patients that had been reached after a mean of 8 months. The time to a nadir RNFL finding and its values were in direct correlation with baseline disease severity. These findings indicate that long-term follow-up of IIH patients, including OCT imaging, is essential for optimal management.

## Data Availability

The datasets used and/or analyzed during the current study are available from the corresponding author upon reasonable request.
