# Author Correction: Longitudinal optical coherence tomography indices in idiopathic intracranial hypertension

**DOI:** 10.1038/s41598-024-61881-y

**Published:** 2024-05-14

**Authors:** Rachel Shemesh, Omry Frige, Sharon Garmider, Ruth Huna-Baron

**Affiliations:** 1 Neuro-Ophthalmology Unit, The Goldschleger Eye Institute, Sheba Medical Center, Tel Hashomer, Israel; 2 Faculty of Medicine, Tel Aviv University, Tel Aviv, Israel; 3 Arrow Project, Sheba Medical Center, Tel Hashomer, Israel

Correction to: *Scientific Reports* 10.1038/s41598-024-58865-3, published online 14 April 2024

The Acknowledgements section in the original version of this Article was incomplete.

“The study was supported by The Claire & Amedee Martier Institute for the Study of the Blindness & Visual Disorders.”

now reads:

“The study was supported by The Claire & Amedee Martier Institute for the Study of the Blindness & Visual Disorders and by Richard Stein Foundation for Ophthalmic Research.”

The original Article has been corrected.

